# Prioritizing feature bindings across space and modality in working memory

**DOI:** 10.3758/s13421-025-01804-y

**Published:** 2025-10-17

**Authors:** Hatice Cinar, Amy L. Atkinson, Amanda H. Waterman, Richard J. Allen

**Affiliations:** 1 School of Psychology, University of Leeds, Leeds, LS2 9JT UK; 2 Department of Psychology, Lancaster University, Lancaster, UK; 3 Department of Psychology, Kirklareli University, Kirklareli, Türkiye

**Keywords:** Working memory, Prioritization, Binding, Unitization, Cross-modality

## Abstract

A growing body of evidence shows that selective attention can be strategically directed to prioritize items of higher “value” in working memory. This work has typically been limited to tests of feature binding using simple “unitized” colored shapes as memoranda. Recent research has suggested prioritization may not be effectively applied to color-odor bindings. This raises the possibility that the benefits of value-based prioritization in working memory may be limited to bindings between unitized visual features (e.g., object-color bindings, object-location bindings). The current study explored this, examining whether value-directed prioritization can be effectively applied when feature pairings of color and shape are separated out, either into different spatial locations (Experiment 1) or across visual and auditory presentation modalities (Experiment 2). Experiment 1 found an overall cost to working memory performance when features were spatially separated, relative to a unitized condition, while Experiments 2 found no such cost for cross-modal feature separation. Across the experiments, participants were equally able to derive performance benefits from prioritizing high-value items in the sequence, regardless of whether features were encountered as part of the same unitized object or separated in space (Experiment 1) or modality (Experiment 2). The findings have implications for the relationship between working memory and attention, suggesting that value-directed prioritization can be effectively applied across different types of feature binding.

**Supplementary Information:**

The online version contains supplementary material available at 10.3758/s13421-025-01804-y.

## Introduction

Our ability to temporarily hold and process environmental input is served by working memory, a capacity-limited system that is integrally connected with broader cognition and closely related to attention (e.g., Baddeley et al., 2021; Cowan et al., 2021). This relationship means that what we attend to determines what we remember in working memory, and vice versa (e.g., Cowan et al., 2024; Oberauer, 2019), a useful feature given the constrained capacity of this system. The interaction between working memory and attention has been empirically demonstrated through experimental methods that promote the prioritization of certain items within a working memory task. For example, a visual cue might be presented before or after encoding that indicates which item is more likely to be tested (e.g., Griffin & Nobre, 2003; Hautekiet et al., 2025; Souza & Oberauer, 2016; Zhang & Lewis-Peacock, 2023). Alternatively, differential value or reward might be associated with memoranda from a sequence or array, with the aim of encouraging participants to strategically prioritize items of higher value (e.g., Hu et al., 2014). The present study focuses on this latter method, examining in two experiments whether value-directed strategic prioritization can be effectively applied to different classes of object.

In the value-directed prioritization paradigm (see Allen et al., 2025, for a review), notional point values are allocated to each item, with participants told they will collect these points if they correctly respond at test. In the pre-presentation methodology that is the focus of the current study, point values are allocated to items prior to their encoding and are not predictive of what is likely to be relevant at the test phase. Compared to an equal value condition with no variation in reward, this instruction typically results in improved memory for the higher value items, some costs distributed across lower value items, and no overall change in performance. Thus, attentional focus can be flexibly shifted between items to ensure some are prioritized, though this does not mediate global working memory capacity. This prioritization effect has been attributed to one or more items being retained in an active and accessible state within working memory. When a sequence of items is encountered, each is normally registered and briefly held in such a state within the focus of attention, which is why an advantage for the most recent item is often observed (e.g., Allen et al., 2006, 2014). However, we can strategically prioritize a particular item during encoding and maintenance, possibly through a process of biased attentional refreshing (Atkinson et al., 2022), to help ensure that it is active and accessible within the focus of attention (Allen et al., 2025; Hitch et al., 2020). This focus of attention is the core aspect of Cowan’s embedded processes model (e.g., Cowan et al., 2021) and has been incorporated as the episodic buffer within the multicomponent model of working memory (Baddeley et al., 2021; Hitch et al., 2025).

Most work on prioritization has been done in the visual domain using tasks requiring memory for the bindings between color and shape when features are encountered as part of the same unitized object (e.g., Allen et al., 2021; Allen & Ueno, 2018; Atkinson et al., 2018, 2019, 2022; 2025; Hitch et al., 2018; Hu et al., 2014, 2016). The core findings have also been extended to photographic objects (Atkinson et al., 2024), visually presented words (Sandry et al., 2014, 2020), visually presented words in spatial locations (Jeanneret et al., 2025), auditory-verbal recall of digits (Atkinson et al., 2021), and sequences of tactile input (Roe et al., 2024), indicating the phenomenon to be modality-general rather than specific to any one task or input stream.

However, there has been very little exploration of whether non-predictive value-directed prioritization can be applied during both encoding and maintenance to other forms of feature binding. One exception to this is a study by Johnson and Allen (2023) that examined memory for short sequences of color-odor bindings (using different odors encountered in colored boxes). Allocation of higher value to the first item in the sequence shifted the profile of performance across serial positions, indicating some attempt to prioritize, but there was no clear evidence that this enhanced accuracy for the high-value item, compared to an equal-value baseline. Thus, prioritization in this context appeared to be relatively ineffective.

This raises the as-yet unexplored question of whether prioritization can be effectively applied across non-unitized forms of binding. A few previous studies have explored memory for separation of feature pairings across space and presentation modality, though not in the context of prioritization (e.g., Allen et al., 2009; Gao et al., 2017; Guazzo et al., 2020; Karlsen et al., 2010; Parra et al., 2015; Wang et al., 2015). Separation by space or modality may represent strong forms of what have been termed “relational binding,” compared to the conjunctive binding involved in visually unitized objects (Parra et al., 2015). Starting with spatial separation, Karlsen et al. (2010) presented shape-color pairings in either unitized form (i.e., as single objects) or with the visual features spatially separated into vertically adjacent locations. Immediate recognition accuracy for feature binding was relatively lower when features were spatially separated, indicating a unitization advantage (see also van Dam & Hommel, 2010; Xu, 2002a, 2002b), but this did not interact with concurrent task condition, suggesting a similar involvement of central executive control resources in each case. As part of the same research series, Allen et al. (2009) contrasted visually unitized binding with a cross-modal condition in which feature pairings were simultaneously presented in visual and auditory modalities. Concurrent load again did not interact with binding condition, and in this case overall recognition accuracy was equivalent for visual and cross-modal combinations. These studies were interpreted as evidence that features encountered across space or modality can be bound together in working memory without necessarily placing additional costs on executive control (see Baddeley et al., 2011, for a review).

According to the object file theory of feature binding (Kahneman et al. 1992), visual features that share spatial location are bound together into an integrated object representation. Al Hadhrami et al. (2025) have recently suggested that multi-feature objects can be held and accessed as either integrated object units or via pairwise connections between individual features, depending on the specific combinations of features and how they are encountered in the environment. Building on this, we assume that when feature pairs are encountered in a non-unitized form (e.g., separated by space or across modalities), they are unlikely to generate integrated object representations, and instead would be indirectly linked and later retrieved via shared presentation time (Bowman & Wyble, 2007; Schneegans et al., 2023). One possibility that then arises is that mechanisms of biased encoding and attentional refreshing within the focus of attention that underlie prioritization effects (e.g., Atkinson et al., 2022) are less effectively applied to these more indirect connections between feature bindings, compared to integrated object representations involved in visually unitized features such as colored shapes.

Therefore, in bringing together the previously separate literatures on prioritization and binding, this may explain why value-based prioritization has been found to be effective in object-based binding tasks (Allen et al., 2025) but may have been relatively ineffective for odor-color bindings (Johnson & Allen, 2023). However, aside from the study of odor-color binding reported by Johnson and Allen (2023), very little is known about how selective prioritization might be applied to binding of features that are not visually unitized in the environment, where perceptual binding as integrated and unitized objects is no longer as plausible. As Johnson and Allen (2023) highlighted, there is a need to determine empirically whether prioritization effects are generally weaker for different forms of binding (e.g., cross-modal, extrinsic, or relational), or whether such limitations are specific to olfactory memory.

We examined this question in two experiments. The first experiment remained focused within the visuospatial domain, comparing working memory for feature bindings when the constituent elements were encountered in a visually unitized form (as in previous research employing value-based prioritization; e.g., Atkinson et al., 2018; Hitch et al., 2018), or were separated into distinct spatial locations. Experiment 2 examined working memory for binding of features that were separated across visual and auditory modalities, again contrasting with unitized binding. In each case, we examined whether prioritization would be less effective when features were separated and only linked via shared presentation time, compared to when they are encountered as visually unitized objects.

## Experiment 1

The first experiment examined whether value-directed strategic prioritization effects would vary with unitization of visual features, asking whether prioritization is more effective for memoranda when constituent features are encountered as single objects rather than spatially distinct feature pairings. Spatial location is an important dimension in supporting binding of visual features (Rajsic & Wilson, 2014; Schneegans & Bays, 2017; Shepherdson et al., 2022; Treisman, 1982; Treisman & Gelade, 1980), and there is some evidence for different neural underpinnings for feature binding when features are not visually unitized as single objects (e.g., Parra et al., 2015). Memory for unitized and spatially separated features was directly compared in a series of experiments by Karlsen et al. (2010). Results indicated reduced recognition performance for binding when features were separated in space, compared to when they were visually unitized as single objects, though this did not interact with concurrent attentional load. Thus, there is a performance cost to working memory binding from separating features in space, but this does not necessarily reflect any greater requirement for executive resource.

The current study used an adapted version of the Karlsen et al. (2010) paradigm to provide the first exploration of strategic prioritization effects in spatially separated feature binding. Each trial involved sequences of four feature pairings presented in either visually unitized (as single objects) or spatially separated (with shape and color as spatially proximate but separate stimuli) forms. Value-directed prioritization was implemented as in related studies (e.g., see Allen et al., 2025), comparing a no-priority condition in which all items were of equal value with a priority condition in which the first item in the sequence was allocated higher value. As our aims in the current study were to establish whether benefits of value-directed prioritization in working memory can be derived for features separated over space (or over modality in Experiment 2), we implemented the same single-item cued recall paradigm that has proved to be reliable in demonstrating such effects in visual object binding (e.g., Allen & Ueno, 2018; Allen et al., 2021; Atkinson et al., 2018, 2019; Hitch et al., 2018; Hu et al., 2014, 2016).

We predicted several effects to emerge in this experiment. Firstly, if the effectiveness or ease of prioritization does vary with binding type and the nature of the underlying representation, these two factors should interact. Specifically, if prioritization of spatially separated feature bindings is less effective, we might expect a relatively smaller boost to the high-value first position, compared to that observed in the unitized condition. Secondly, we expected any prioritization effects to emerge in the context of no overall effect of prioritization condition across all trials, as prior work indicates that this manipulation does not enhance overall working memory capacity (Allen et al., 2025). Finally, based on Karlsen et al. (2010), we predicted lower performance for spatially separated features relative to the unitized condition, reflecting how the former condition requires the encoding and association of two visual feature dimensions encountered in distinct spatial locations.

### Method

#### Participants

Estimated power and appropriate sample size was calculated using Superpower (Lakens & Caldwell, 2021), focusing on the key analysis of interest (the 2 × 2 ANOVA at the targeted SP (SP1)). No prior studies have examined priority effects on spatially separated and cross-modal binding. Power and sample size estimates were therefore calculated using means and SE values drawn from Karlsen et al., (2010, Experiment 2) for the main effect of binding (unitized vs. spatially separated), and Atkinson et al., (2018, Experiment 1) for the effect of main effect of prioritization (no priority vs. priority-SP1). As in the present work, these earlier studies involved single-item tests of memory of sequences of four colored shape combinations. For the interaction, we predicted that the prioritization effect in the spatially separated condition would be smaller than that in the unitized condition. As a reasonable estimate of this predicted reduction, we based our power calculation on the prioritization effect in the spatially separated condition being half the size of the effect observed in the unitized case in Atkinson et al., (2018; Experiment 1). Based on alpha =.05 and 80% power, this indicated a sample size of 32 was required to detect main effects of binding (estimated partial eta squared =.73, Cohen”s f = 1.63) and prioritization (.71, 1.58), and an interaction between binding and prioritization (.22,.53).

Thirty-five participants completed the experiment (aged 18–30 years; *M* = 20.3; *SD* = 2.2; 27 females, seven males and one other), taking part either for course credit or no reward. They were all native English speakers, and none reported a history of neurological disorders. The participants had normal or corrected-to-normal vision and no color blindness. Informed consent was acquired in accordance with the guidelines set by the University of Leeds’ Psychology Ethics Committee (Ethics reference number: PSC-325 and PSCETHS-1020).

#### Materials

Six colors (black, red, blue, green, yellow, and purple) and six shapes (circle, cross, diamond, star, flag, and triangle) were used as visual stimuli, as taken from Allen et al. (2006). A neutral formless shape (“a blob”) and shape outline of the same six shapes were utilised to display colors in spatially separated conditions and present as a test cue (Allen et al., 2009). Shapes and colors were not repeated within the same trial. All stimuli were presented in size 3.3 × 3.3 cm (124.72 × 124.72 pixels) based on a standard small monitor screen (1,280 × 1,024 pixels), (33.5 ×  27 cm) on a white background and viewed from a distance of approximately 50 cm.

#### Design and procedure

A 2 × 2 repeated-measures design was implemented in each experiment, with two types of binding type (unitized and spatially separated) and two prioritization conditions (priority-SP1 and no-priority).

The Gorilla Experiment Builder (www.gorilla.sc) was used to create the experiment and collect data (Anwyl-Irvine et al., 2020), with the experimental session conducted in person. Participants completed four blocks (one per condition—combination of binding type and prioritization) and each block included 40 test trials. Order of condition blocks was fully counterbalanced across participants. Each serial position was tested an equal number of times (ten times) in a random order within each block. There were two practice trials at the beginning of each block to familiarize participants with the condition.

At the beginning of all conditions, participants were informed of task details via written instructions. In the prioritization condition, they were told that the first stimulus would be paired with 10 points while the other three stimuli were worth 1 point. The precise instruction was “The first pair is going to be worth 10 points if you are tested on it and get it correct. The other items are worth 1 point. So, try to especially focus your attention on the first pairing as this will be worth more points.” In the no-priority condition, participants were informed that all stimuli were paired with 5 points. The precise instruction for no-priority condition was “Each item is going to be worth 5 points if you are tested on it and get it correct.” Thus, in the no-priority condition, none of the items were explicitly to be prioritized, whereas in the priority-SP1 condition, the first stimulus was to be prioritized. Point values were notional and were not predictive of which item would be tested.

**Fig. 1:**
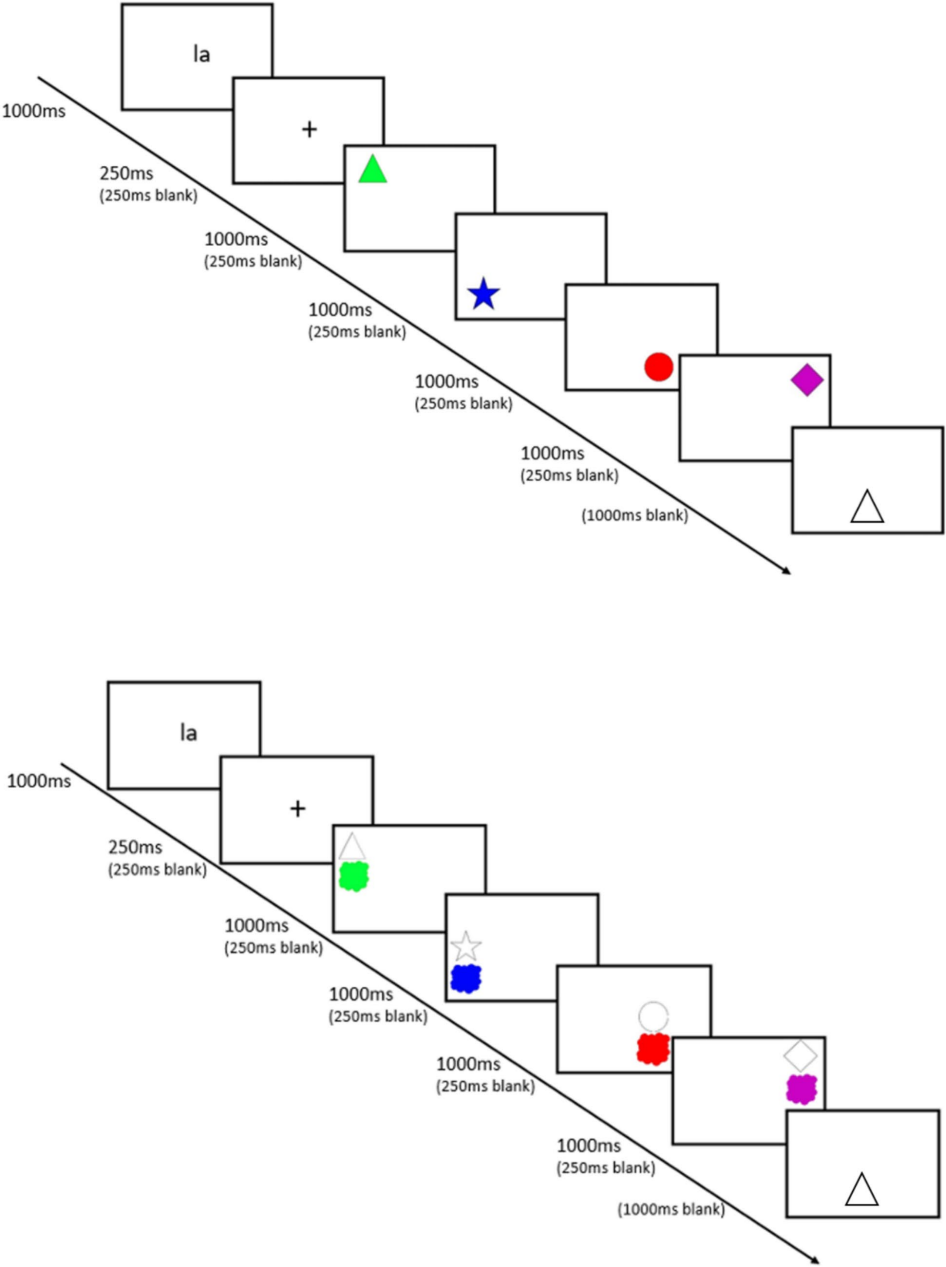
The experimental paradigm used in Experiment 1. The top panel shows a unitized trial, whilst the bottom panel shows a spatially separated trial. Figure not to scale

There were two different binding conditions. In the unitized condition, colors and shapes were presented as a single-colored shape (e.g., a circle outline with red infill). In the spatially separated condition, colors and shapes were presented simultaneously but visually separated as pairs of colored blobs and unfilled shapes (e.g., a red-colored blob and the outline of a circle). In this condition, colors and shapes were displayed as vertically adjacent, with colors always presented directly below the shapes, separated by 0.6 cm (see Fig. 1).

Figure 1 shows the experimental paradigm. To-be-remembered stimuli were presented in the four corner quadrants of an imaginary rectangle (26.8 × 17.26) cm in a standard small monitor screen (1,280 × 1,024 pixels), in pseudo-randomizing position with the constraint that each location was only occupied once per trial and in a counterbalanced order. For example, the first shape color pairing was shown in the upper-right corner, the second in the lower-left corner, and so on. In each trial, only one item was presented within each quadrant.

Each trial began with the 1,000-ms presentation of the nonword "la" which participants were asked to repeat until the retrieval phase to disrupt verbal rehearsal. Adherence was monitored by a researcher, who remained in the room for the duration of the testing session. A fixation cross then appeared at the center of the screen for 250 ms, followed by a 250-ms blank screen. Each of the four visual stimuli was presented on a white background in one of the four corner quadrants of the screen for 1000 ms with an inter-stimulus interval of 250 ms. A 1000 ms blank screen delay followed the presentation of the four stimuli then the test cue was presented. The test cue, a shape outline, was pseudo-randomly selected from the four stimuli in the study array with the restriction that each SP was tested an equal number of times within each condition per participant. The test cue was presented below the screen center so as not to spatially overlap with the target. Participants were asked to verbally recall the name of the color that was presented with that shape. The experimenter recorded their answers and then pressed the enter button to progress to the next trial. Reminders about the item values were presented to participants after every 20 trials. Participants were given feedback on their ongoing points score halfway through each block, and their total points score at the end of each block.

### Data analysis

Data for both experiments are available on the Open Science Framework (https://osf.io/6yu84). The outcome variable for Experiments 1 and 2 was proportional accuracy in recalling the correct color. The independent variables were binding type (unitized and spatially separated in Experiment 1; unitized and cross-modal binding in Experiment 2) and prioritization (priority-SP1 and no priority). Our analytic approach followed that of Atkinson et al. (2025). Thus, the data were subjected to a 2 (binding types) × 2 (prioritization) repeated-measures ANOVA. Results are initially reported in terms of the effect at SP1 as this SP is targeted in prioritization. Additional planned analysis was conducted at lower value SPs (positions 2–4) to explore any effects of prioritizing SP1 on these low value items elsewhere in the sequence. Finally, we also implemented two paired-samples t-tests examining performance on all trials, firstly comparing the two priority conditions, and secondly comparing the two binding conditions, to establish whether prior observations (e.g., Hitch et al., 2018; Karlsen et al., 2010) for these two factors were replicated at a global level. Note that we also conducted a three-way ANOVA (a 2 [priority condition] × 2 [binding type] × 4 [serial position]) ANOVA to examine performance across all SPs within the same analysis, reported in the Online Supplementary Materials, though outcomes from this analysis should be interpreted with caution given our power calculation was based on the main 2 × 2 ANOVA targeted at the first serial position.

Data analysis was conducted using frequentist and Bayes Factor (BF) methods, using JASP (Version 0.16). Our Bayesian analysis considers the likelihood of the data under both the null and alternative hypotheses, compared via the Bayes Factor (*BF*). A *BF* between 1 and 3 is considered to reflect anecdotal evidence, between 3 and 10 indicates moderate evidence, and a *BF* of 10 or above is considered strong evidence (Jarosz & Wiley, 2014; Schönbrodt & Wagenmakers, 2018).

### Results

#### Effect at SP1 (targeted SP)

**Fig. 2:**
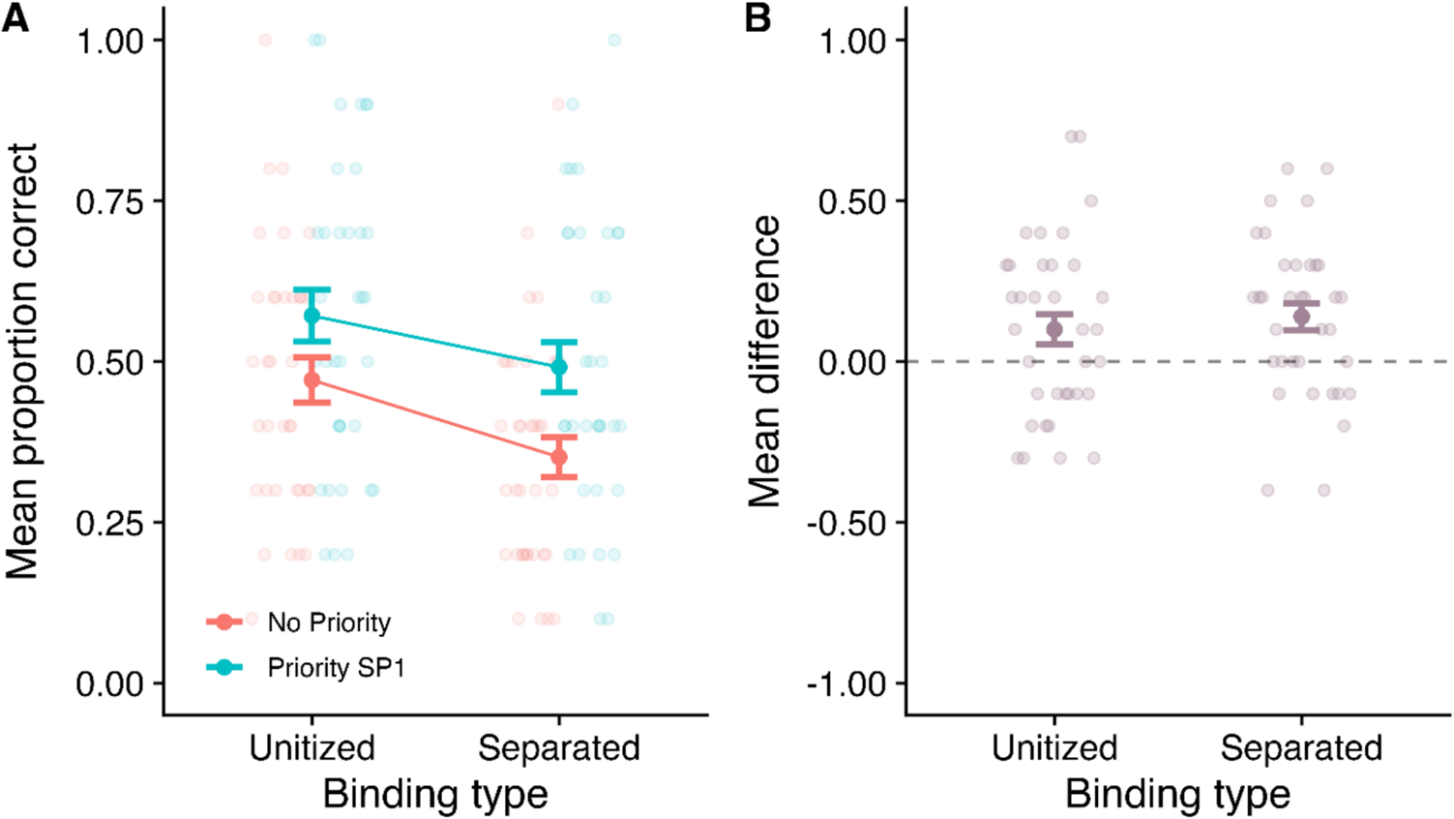
(**A**) Mean performance at serial position 1 for the priority and binding conditions. (**B**) Mean difference between priority-SP1 and no-priority conditions at serial position 1 for each binding type. Values above 0 indicate higher performance in the priority-SP1 condition. Error bars show SE and light dots show individual participants

Figure 2 shows mean performance at serial position 1 (the targeted SP) in the binding and priority conditions, along with the mean difference in performance between priority-SP1 and no-priority. A 2 (Priority: Priority SP1 vs. no-priority) × 2 (Binding type: unitised vs. separated) repeated-measures ANOVA revealed a main effect of prioritization (*F*(1,34) = 13.92, *p* <.001, ${\eta }_{p}^{2}$ =.29; *BF*_*10*_ = 35.61), with higher accuracy in the priority-SP1 (M =.53, SE =.03) than in the no priority condition (M =.41, SE =.03). There was also a main effect of binding type (*F*(1,34) = 14.97,* p* <.001, ${\eta }_{p}^{2}$ =.31; *BF*_*10*_ = 15.04); performance was higher for unitized (M =.52, SE =.03) than spatially separated binding (M =.42, SE =.03). There was no significant interaction between prioritization and binding type (*F*(1,34) =.44, *p* =.513, ${\eta }_{p}^{2}$ =.01; *BF*_*10*_ =.31), indicating that performance was enhanced for the first item in the priority SP1 compared to no-priority condition to an equivalent extent in the two binding conditions.

#### Effects on less valuable SPs (2–4)

**Fig. 3:**
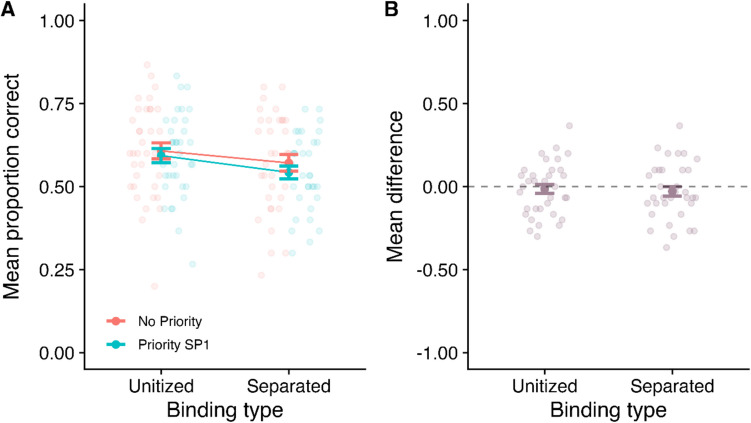
(**A**) Mean performance averaged across low value serial positions (2–4) for the priority and binding conditions. (**B**) Mean difference between priority-SP1 and no-priority conditions across low value serial positions (2–4) for each binding type. Values below 0 indicate lower performance in the priority-SP1 condition. Error bars show SE and light dots show individual participants. Here, “Priority-SP1” refers to the experimental condition in which SP1 was assigned high value, but the data in the panels reflect the low-value positions (SP2–SP4)

Figure 3 shows mean performance averaged across low value serial positions (2–4) in the binding and priority conditions, along with the mean difference in performance between priority-SP1 and no-priority. A 2 (Priority: Priority-SP1 vs. no-priority) × 2 (Binding type: unitised vs. separated) repeated-measures ANOVA revealed a main effect of binding (*F*(1, 34) = 5.55, *p* =.024, ${\eta }_{p}^{2}$ =.14; *BF*_*1*0_ = 1.57), with higher accuracy in the unitized (*M* =.60, *SE* =.02) than in the spatially separated condition (*M* =.56, *SE* =.02), though the Bayesian evidence was weak. There was no main effect of prioritization (*F*(1, 34) = 1.05, *p* =.314, ${\eta }_{p}^{2}$ =.03; B*F*_*10*_ =.36). There was also no significant interaction between prioritization and binding type (*F*(1, 34) =.16, *p* =.697, ${\eta }_{p}^{2}$ =.01; *BF*_*10*_ =.26).

#### Overall differences between priority and binding conditions

Finally, we examined whether overall performance (across all serial positions) varied with binding condition and prioritization. A pair of paired-sample t-tests was conducted, comparing unitized versus spatially separated binding, and Priority-SP1 versus No-Priority. As predicted, a significant difference emerged between separated and unitized conditions, with significantly higher accuracy in the unitized condition (*M* =.58, *SE* =.02) than in the spatially separated condition (*M* =.52, *SE* =.01), (*t*(34) = 3.86,* p* <.001, *BF*_*10*_ = 59.97, *d* =.65). Also as predicted, there was no overall difference in performance (*t*(34) =.89,* p* =.378, *BF*_*10*_ =.26, *d* =.15) between priority-SP1 (*M* =.56, *SE* =.01) and no-priority conditions (*M* =.55, *SE* =.02).

### Discussion

This experiment provides the first observation of a strategic value-directed prioritization effect in memory for visual features that are separated in space. For both visually unitized and spatially separated feature pairings, we found a benefit of value-directed prioritization for the higher value items presented at serial position 1 compared to an equal value condition, in the context of no overall change in performance (e.g., Atkinson et al., 2018; Hitch et al., 2018; see also Allen et al., 2025). Small numerical costs were observed on less valuable items, but these were not supported by statistical analysis. Importantly, both the frequentist and Bayes factor outcomes were clear in indicating a null effect for the interaction between binding type and priority, when analysing performance at the targeted serial position (SP1), or for items at other positions in the sequence. Thus, strategic prioritization was applied in an equally effective way regardless of whether the features were encountered as single visually unitized objects or separated in space. Rather than indicating any greater difficulty in applying focused attention to non-unitized relational feature bindings, this finding suggests that prioritization can be equally applied to these distinct types of binding. This observation draws parallels with the previous observation of similar concurrent attentional load effects on spatially separated and unitized binding (Karlsen et al., 2010). These findings regarding prioritization were observed in the context of a replication of the previously observed unitization advantage in visual working memory (Karlsen et al., 2010), with improved recall for features presented as part of the same visual object rather than being separated in space.

## Experiment 2

The first experiment indicated that whilst spatial separation of visual features results in an overall reduction in performance, it does not lead to any measurable change in the effectiveness of strategic prioritization. Thus, the form of relational binding that might underlie memory for spatially separated visual features can be encoded into the focus of attention and held in an active and accessible form, potentially through attentional refreshing (Allen et al., 2025; Atkinson et al., 2022), as effectively as conjunctive object-based representations that might underlie memory for visually unitized colored shapes. It is important to test the generality of these observations for other forms of relational binding using the same paradigm. Experiment 2 aimed to explore beyond the visuospatial domain, testing whether the same principle applies when to-be-remembered feature pairings are separated by encoding modality. As noted by Arslan et al. (2025), recent working memory research has strongly focused on the visual domain, with limited understanding concerning how multisensory objects are temporarily encoded and maintained. Cross-modal binding may be a particularly useful test of a modality-general working memory component such as the episodic buffer or focus of attention, as the different input streams would not be captured within the same specialized subsystem (Allen et al., 2009; Wang et al., 2015, 2017). Performance in the spatially separated condition (as in Experiment 1) might still be achieved externally to the episodic buffer, for example, within the visuospatial sketchpad (Baddeley et al., 2021), based on separately stored visual features that share ordinal and timing signals along with proximate spatial location. In contrast, cross-modal binding can only be achieved within the episodic buffer (Baddeley et al., 2021), or the focus of attention within Cowan’s embedded processes approach (e.g., Cowan et al., 2021). The same would apply to olfactory-color binding, which appears to be possible in general, though with limited capacity and scope for prioritization of particularly valuable pairings (Johnson & Allen, 2023). Actively prioritizing cross-modal bindings may therefore be more challenging, as processing of each new pairing that is encountered might detract from the prioritization of an earlier item within the episodic buffer.

Experiment 2 adapted the paradigm implemented by Allen et al. (2009), and later used by Guazzo et al. (2020), in which a visual feature (in this case, shape) is paired with an auditory feature (color name), to provide the first examination of strategic prioritization of cross-modal binding. Using single probe recognition, Allen et al. (2009) found that accuracy for cross-modal binding was equivalent to that observed in unitized binding, showing no performance decline (or benefit) from presenting across, rather than within, modalities. This stood in contrast to reduced accuracy for spatially separated binding observed by Karlsen et al., (2010; see Experiment 1). Similarly to Karlsen et al., dual-task manipulations indicated no increased reliance on attentional control for cross-modal binding (Allen et al., 2009), suggesting that executive control resources are not particularly necessary during encoding and maintenance. In line with this, Arslan et al. (2025) have recently demonstrated that encoding and maintenance of visual-auditory binding can occur in a relatively automatic, bottom-up manner. This would also fit with Guazzo et al. (2020), who applied a cued-recall task and found that cross-modal binding was no more affected than unitized binding by healthy aging or Alzheimer’s disease.

In this experiment, we again predicted a prioritization-based recall enhancement of the high-value item relative to an equal value baseline, along with small costs to recall of low-value items and no overall effect of priority condition across all trials. Critically, we tested the hypothesis that feature bindings encountered in different modalities are more difficult to effectively prioritize (Johnson & Allen, 2023). Based on this, we predicted that cross-modal binding should show a reduced prioritization benefit relative to the unitized condition. Alternatively, a null interaction would align with outcomes from Experiment 1 and show that prioritization can be just as effective cross modally as within modality. Finally, based on previous findings (Allen et al., 2009; Guazzo et al., 2020), we predicted no overall difference in accuracy between the two binding type conditions (unitized and cross-modal), extending such observations from single probe recognition (Allen et al., 2009) to cued recall in the present paradigm.

### Method

#### Participants

Estimated power and sample size was closely based on the method used in Experiment 1, but this time predicting no difference between unitized and cross-modal binding in the no-priority condition (based on Allen et al., 2009). This again indicated a sample size of 32 was required to detect the main effects of binding (partial eta =.22, Cohen’s f =.53) and prioritization (.71, 1.58), and the interaction between binding and priority condition (.22,.53) in the primary analysis examining performance at serial position 1.

Thirty-five participants (aged 18–22 years; *M* = 19.2; *SD* =.9; 30 females and five males) took part in this experiment in exchange for course credit or no reward. They were all native English speakers, and none reported a history of neurological disorders. The participants had normal or corrected-to-normal vision and no color blindness. Informed consent was acquired in accordance with the guidelines set by the University of Leeds’ Psychology Ethics Committee (Ethics reference number: PSYC-608 and PSCETHS-1020).

#### Design and procedure

**Fig. 4:**
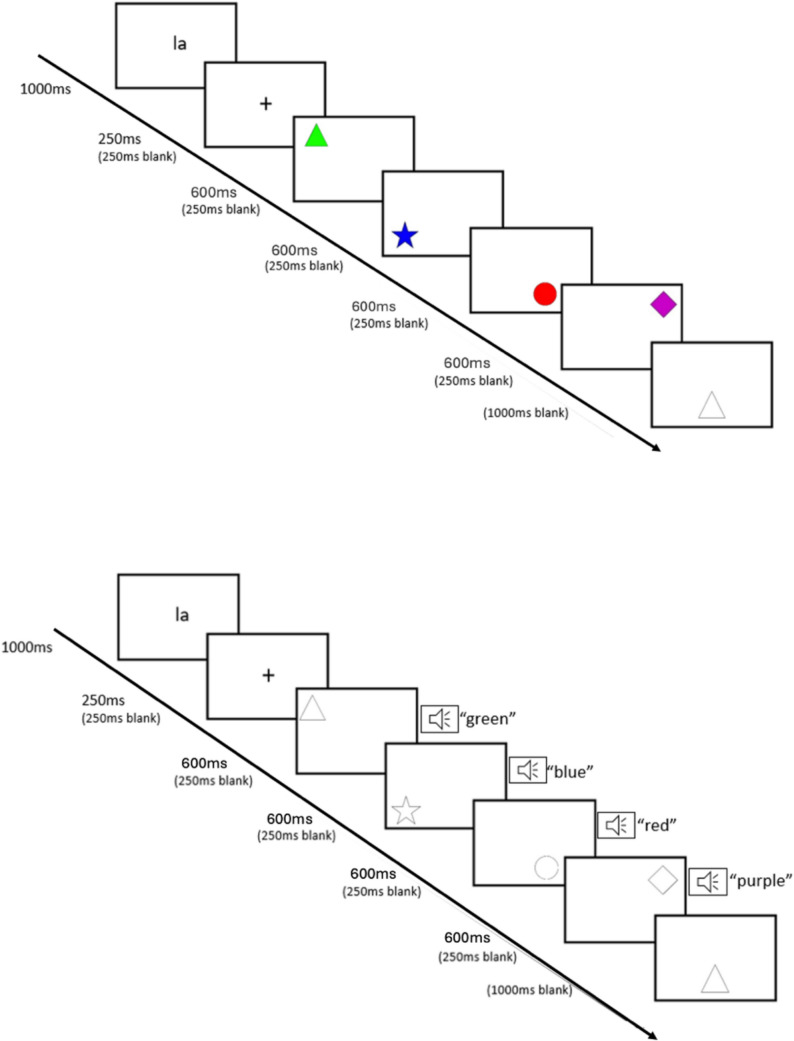
Illustration of the paradigm used in Experiment 2

The method was closely based on Experiment 1, with the same material set, design, and trial procedure, but comparing visually unitized and cross-modal feature binding. In the cross-modal binding condition, each shape was presented visually in pairing with an auditory color name (see Fig. 4). Auditory stimuli consisted of the six color names spoken by a digitized speaker using a female English-accented voice. As these stimuli were naturally voiced at between 450 and 600 ms in duration, it was important to ensure synchrony between visual and auditory exposure.^1^ Therefore, stimulus exposure was adjusted to 600 ms per item pairing for both the unitized and cross-modal conditions in this experiment. This is comparable to exposure times used in a range of previous studies examining prioritization of unitized feature pairings (e.g., 500 ms per item in Atkinson et al., (2018, 2019) and Hu et al. (2023)). All other inter stimulus interval times were kept consistent with Experiment 1.

Four paired visual and audio stimuli in each trial were serially presented, after which the visual test probe followed. The test probe was always a shape and provided in the visual modality, and as in Experiment 1 participants needed to verbally recall the name of color that was paired with the shape.

As with Experiment 1, a 2 × 2 repeated-measures design was implemented in each experiment, with two types of binding type condition (unitized and cross-modal binding) and two types of prioritization condition (priority-SP1 and no-priority).

### Results

#### Effect at SP1 (targeted SP)

**Fig. 5:**
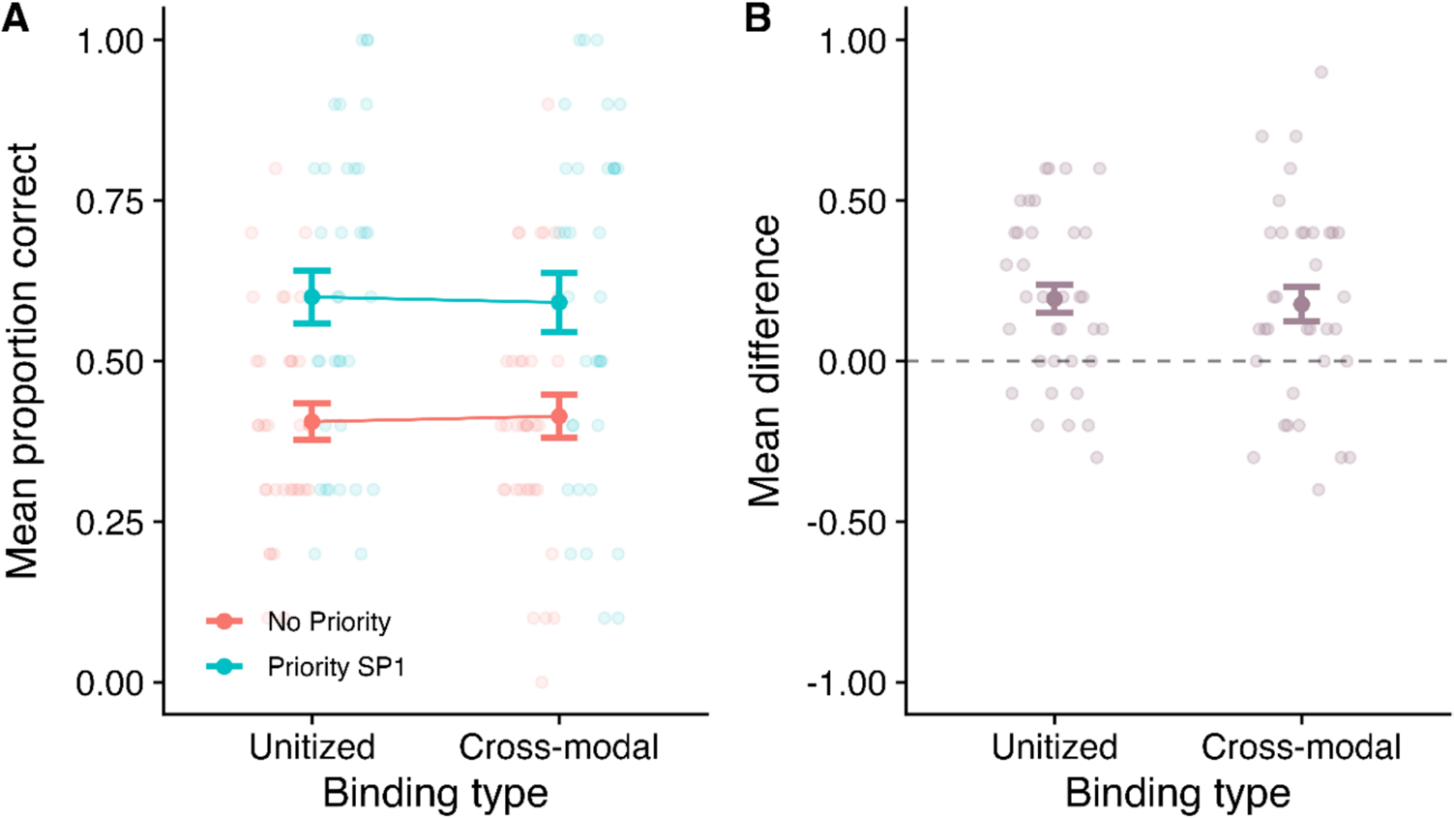
(**A**) Mean performance at serial position 1 (the targeted SP) for the priority and binding conditions. (**B**) Mean difference between priority-SP1 and no-priority conditions at serial position 1 for each binding type. Values above 0 indicate higher performance in the priority-SP1 condition. Error bars show SE and light dots show individual participants

Figure 5 shows mean performance at serial position 1 in the binding and priority conditions, along with the mean difference in performance between priority-SP1 and no-priority. A 2 (Priority: Priority SP1 vs. no-priority) × 2 (Binding type: unitised vs. cross-modal) repeated-measures ANOVA revealed a main effect of prioritization (*F*(1, 34) = 21.18, *p* <.001,  ${\eta }_{p}^{2}$ =.38; *BF*_*10*_ = 474.68), with higher accuracy in the priority-SP1 condition (*M* =.60, *SE* =.04) relative to the no-priority condition (*M* =.41, *SE* =.02). There was no main effect of binding type (*F*(1, 34) <.001, *p* = 1.00, ${\eta }_{p}^{2}$ <.001; *BF*_*10*_ =.25), and no interaction between prioritization and binding type (*F*(1, 34) = 0.09, *p* =.763, ${\eta }_{p}^{2}$ = 0.003; *BF*_*10*_ = 0.22), indicating that there was an improved performance in the priority-SP1 compared to the no-priority condition, and this improved performance in the priority condition did not differ depending on the binding type.

***Effects on less valuable SPs (2***–***4).***

**Fig. 6:**
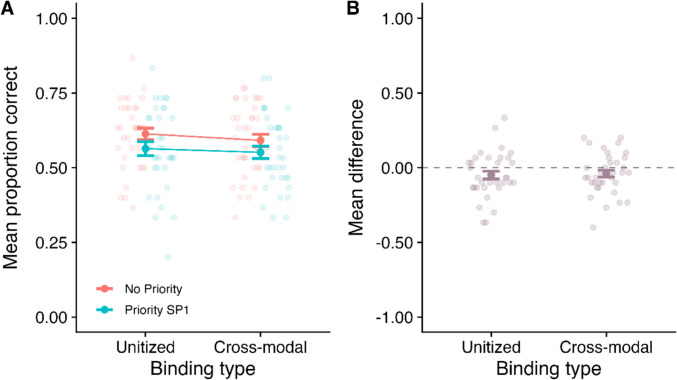
(**A**) Mean performance averaged across low value serial positions (2–4) for the priority and binding conditions. (**B**) Mean difference between priority-SP1 and no-priority conditions for each binding type. Values below 0 indicate lower performance in the priority-SP1 condition. Error bars show SE and light dots show individual participants. Here, “Priority-SP1” refers to the experimental condition in which SP1 was assigned high value, but the data in the panels reflect the low-value positions (SP2–SP4)

Figure 6 shows mean performance averaged across low value serial positions (2–4) in the binding and priority conditions, along with the mean difference in performance between priority-SP1 and no-priority. A 2 (Priority: Priority-SP1 vs. no-priority) × 2 (Binding type: unitised vs. separated) repeated-measures ANOVA revealed a main effect of prioritization(*F*(1, 34) = 6.55, *p* =.015, ${\eta }_{p}^{2}$ =.16; *BF*_*10*_ = 2.21), with higher accuracy in the no-priority (*M* =.60, *SE* =.02) than the priority-SP1 condition (*M* =.56, *SE* =.02), though the Bayesian evidence here was only weak. There was no main effect of binding (*F*(1, 34) =.85, *p* =.362, ${\eta }_{p}^{2}$ =.02; *BF*_*10*_ =.32). There was also no significant interaction between prioritization and binding type (*F*(1, 34) =.08, *p* =.784, ${\eta }_{p}^{2}$ =.002; *BF*_*10*_ =.25).

#### Overall effects of priority and binding conditions

Finally, we examined whether overall performance (across all serial positions) varied with binding condition and prioritization. A pair of paired-sample t-tests was conducted, comparing unitized versus cross-modal binding, and Priority-SP1 versus No-Priority. As predicted, there was no effect in either t-test, with no significant difference between unitized (*M* =.57, *SE* =.02) and cross-modal binding (*M* =.55, *SE* =.02) (*t*(34) =.72,* p* =.474, BF_10_ =.23, *d* =.12), or between priority-SP1 (*M* =.57, *SE* =.02) and no-priority conditions (*M* =.55, *SE* =.01) (*t*(34) =.76,* p* =.450, BF_10_ =.24, *d* =.13).

### Discussion

The results of Experiment 2 provided novel evidence for a value-directed prioritization effect in working memory for cross-modal binding, that was equivalent in size to the one observed in visually unitized binding. Thus, participants can effectively allocate selective attention to feature pairings when they are separated across visual and auditory modalities. As with Experiment 1, this draws parallels with previously reported evidence of equivalent concurrent attentional load effects across unitized and cross-modal conditions (Allen et al., 2009). Experiment 2 also replicated previous observations that cross-modal binding is as accurate as visually unitized binding, supported by both frequentist and Bayesian analyses (Allen et al., 2009; Guazzo et al., 2020). This differs from the reduced accuracy observed in spatially separated binding (Experiment 1, and Karlsen et al., 2010) and indicates that not all forms of feature separation are equivalent, though the benefits of prioritization appear to be, at least for those examined in the current study.

### General discussion

The present study provided the first test of whether strategically directed selective attention can be applied to feature binding in working memory when features are visually unitized or separated over space or modality. Experiment 1 showed that spatially separated feature pairings could be strategically prioritized just as effectively as visually unitized pairs, even though they were less accurately recalled overall. Experiment 2 then demonstrated that separating features into sequences of pairings encountered in distinct presentation modalities again did not impinge on the ability to strategically prioritize one of these feature pairings. In all cases, cued recall was more accurate for higher value feature pairings, compared to equal value control conditions. Thus, selective prioritization is not limited to unitized binding and can be effectively applied within sequences of feature pairings that are separated in space or modality. These novel findings therefore indicate that the limited benefits of value-directed prioritization observed by Johnson and Allen (2023) in olfactory-color binding may be associated with that specific combination of modalities, and particularly the requirement to handle olfactory information, rather than being more broadly indicative of any form of non-unitized binding. For unitized, spatially separated, and cross-modal binding, directed prioritization enhanced memory for high-value items while not affecting overall performance, providing further support for a limited resource that can be flexibly and strategically shifted around within a working memory task without changing global capacity (Allen et al., 2025; Atkinson et al., 2018; Hitch et al., 2018).

While debate continues between proponents of leading theoretical models of working memory, most agree that attentional control is important in determining what is remembered and what is forgotten (Byrnes & Miller-Cotto, 2023). Indeed, attentional control has been identified as the key predictive factor underlying the involvement of working memory in broader cognition (e.g., Draheim et al., 2022; Mashburn et al., 2021). Strategic prioritization of some information over others is an important component of attentional control, and leading models generally agree on the importance of capturing how different kinds of information are actively and accessibly held in working memory in this way. The interplay between strategic control and automatic capture that plays out in the current paradigm can be broadly captured within high-level working frameworks such as the multicomponent model (Baddeley et al., 2021; Hitch et al., 2025) and the embedded processes approach (Cowan et al., 2021). Within these frameworks, the content of the episodic buffer or focus of attention is assumed to shift from one item to the next as they are encountered via sequential presentation, in a relatively automatic way (Allen et al., 2025). When a higher value stimulus pairing is encountered, participants can strategically direct their attention to it to ensure it remains active and accessible within this state. This is likely to take place during encoding of the pairing and during maintenance via a mechanism such as attentional refreshing (Atkinson et al., 2022; Camos et al., 2018; Raye et al., 2002; Sandry et al., 2020). This appears to be applicable just as effectively when shape and color features are separated into distinct spatial locations or into visual and auditory modalities, compared to visually unitized objects. In that regard, the current pattern of findings fit with the absence of interactions between binding type and concurrent task that have previously been observed, suggesting equivalent executive control involvement across unitized, spatially separated, and cross-modality binding (Allen et al., 2009; Karlsen et al., 2010; see also Baddeley et al., 2011).

Spatially separated and cross-modal binding can each be classed as forms of relational binding (Parra et al., 2015). This type of binding might be maintained indirectly as an associative connection between distinct features, while visually unitized visual pairings are conjunctive in nature and would be more likely to be encoded, maintained, and retrieved as an integrated object representation. In the case of the spatially separated condition in Experiment 1, features were not part of the same visual object and did not share the same exact spatial code. Feature integration theory highlights spatial location as key in initial feature binding (Treisman, 1982; Treisman & Gelade, 1980). Spatial coding is important in visual unitization, and spatial location plays a role in binding object features together (Rajsic & Wilson, 2014; Schneegans & Bays, 2017; Shepherdson et al., 2022). The spatial separation of features in Experiment 1 (and Karlsen et al., 2010) means that this shared spatial location is lacking, preventing full unitization as a single object-based representation. Common contextual timing signals based on position and/or time (Brown et al., 2000; Burgess & Hitch, 1992, 1999; Farrell, 2008) would still be available to link the feature pairings together (Schneegans et al., 2023) but would be unlikely to support object-based storage.

Separated color and shape may then be stored as distinct but connected items in working memory, for example in the visuospatial sketchpad, within the multicomponent framework of working memory (Baddeley et al., 2021; Hitch et al., 2025). This would then represent a greater load on visual working memory, as it involves storage of multiple visual features that compete for capacity (Wheeler & Treisman, 2002) within the same working memory component, without an integrated representation at the object level. This would then explain why there was an overall decrement in performance in the spatially separated condition. The observation of effective prioritization of items in the context of these lower overall accuracy levels indicates that ease or effectiveness of strategic prioritization does not necessarily vary with broader accuracy or task difficulty (though it can become more important under greater working memory load, e.g., Atkinson et al., 2019). This stimulus-based observation is somewhat analogous to group difference observations in Allen et al. (2021), where older adults were relatively less accurate on a visual working memory task compared to a younger group, but just as able to prioritize high-value items from the sequence.

For cross-modal binding, contextual and ordinal timing signals (e.g., Farrell, 2008; Schneegans et al., 2023) may also be important in supporting binding between visual and auditory input. As participants were exposed to visual and auditory stimuli simultaneously in our experiments, this might help give rise to relatively automatic binding, which would also make prioritization more straightforward. Under the multicomponent approach (Baddeley et al., 2021), the separation of visual and auditory features across modalities would draw on distinct specialized subcomponents (i.e., the visuospatial sketchpad and phonological loop), helping explain why no overall decrement in performance is observed relative to unitized binding. This information from different modalities would be stored and become simultaneously available in conscious awareness within the episodic buffer or focus of attention. Prioritization would then be applied within this modality-general format, as is assumed to be the case with any form of single or multimodal stimulus. Note that this description does not critically rest on accepting any one preferred working memory framework; most leading approaches assume activation of distinct capacities for visual and auditory information, with their association or combination captured in modality-general conscious awareness (e.g., Baddeley et al., 2021; Barrouillet & Camos, 2021; Cowan et al., 2021).

The current findings offer intriguing possibilities regarding the nature of feature binding and the capacity of the focus of attention. We have assumed that feature pairings that are separated in space or modality are stored as distinct but linked forms, rather than as fully object-based representations (as might be available for unitized feature pairings). If so, their effective prioritization suggests a multi-item capacity for actively holding and refreshing information in this state, at least for input that is sufficiently dissimilar and simultaneously encountered. Using Cowan’s terminology (e.g., Cowan et al., 2024), attentional control can be effectively applied to ensure that pairs of features are actively maintained within the scope of attention. This is in line with other evidence (e.g., Allen & Ueno, 2018; Hitch et al., 2018; Souza & Oberauer, 2016; Ueno & Allen, 2025) suggesting that “more than one item, but probably less than four” (Cowan et al., 2024, p.190) can be concurrently prioritized in working memory. Alternatively, the notion of a single-chunk capacity still applies if unitized and separated feature bindings have the potential to be held in an integrated object-based form. There is currently little clear evidence regarding the underlying nature of spatially separated or cross-modal representations. In one of the few other studies on this question, Arslan et al. (2025) found evidence that audio-visual feature pairings of tone and shape were bound together in working memory in a bottom-up manner, but observed no indication from behavioral measures or oscillatory activity (using EEG) for full multisensory integration at least during maintenance. Ultimately, further investigation is required to more conclusively establish how different forms of feature separation influence the type of bound representation that is generated and maintained in working memory, and the implications of this for the focus of attention.

Finally, we note that the differential value condition (priority-SP1) used in the present work applied values of 1 and 10 points to low and high-value items, respectively. While this relative contrast slightly differs from the value levels that have more typically been applied in prior work, which has more frequently employed a 1- versus 4-point comparison (Allen et al., 2025), there have been exceptions to this. For example, Atkinson et al. (2025) employed a 1- versus 5-point contrast in their study of unitized feature binding in working memory, and 1- versus 10-point contrasts have been used to examine selective value-directed prioritization in the context of long-term memory (Yin et al., 2021, 2024). Although relatively higher point values yield larger priority effects when varied within a single trial (e.g., Allen & Ueno, 2018; Hu et al., 2014), little is known about whether this would also apply when varied across different conditions. It would be worthwhile to explore whether participants would be more motivated to apply selective attention to higher value items when a vastly greater associated reward is applied (e.g., conditions of 1 vs. 100 points, and 1 vs. 5 points). Nevertheless, this question does not affect the main conclusions that can be drawn from the current work, which were focused on the presence and magnitude of priority effects in unitized versus separated (Experiment 1) or cross-modal (Experiment 2) binding. Within each of these binding conditions, values of 1 and 10 points were consistently used for low and high-value items respectively. If prioritization of separated or cross-modal binding were less effective compared to unitized binding, priority effects should be smaller regardless of the differential point values that were used. Additionally, as in other studies using varying relative point values, there was no overall main effect of priority condition. That is, memory accuracy was equivalent when comparing overall performance in the no-priority vs. prioritise-SP1 conditions. This replicates the observation that value serves to encourage strategic allocation of a fixed resource to certain items and away from others (rather than change overall motivation in the task). It therefore shows that implementation of 10 point-rewards did not impact overall motivation, just as has been shown with research implementing 4-point values. This clearly demonstrates that the same fundamental processes are at play in each case.

### Conclusions

The current study shows that strategic prioritization can be just as effectively applied to feature pairings when they are visually unitized or when constituent features are separated either visually in space (Experiment 1) or across visual and auditory modalities (Experiment 2). Thus, strategic direction of selective attention during encoding and biased attentional refreshing during maintenance, both of which are assumed to underlie the prioritization effect in working memory, are effective when applied to distinct feature pairings that are not initially encountered as single objects. Together, these findings extend the understanding of value-based prioritization by showing that its benefits generalize beyond simple visual contexts to include spatial separation and multi-modal (visual–auditory) bindings, highlighting its robustness in supporting the complex structure of working memory.

## Supplementary Information

Below is the link to the electronic supplementary material.

Supplementary file1 (DOCX 1289 kb)

## Data Availability

All data and research materials are available at https://osf.io/6yu84/

## Footnotes

1 An initial experiment (N = 30) was carried out that featured this asynchrony, yielding somewhat unclear outcomes. The results are summarised in the Online Supplementary Materials.

## Declarations

### Open Practices Statement

This study’s design and its analysis were not pre-registered.
