# Supplementary material for: Prioritizing feature bindings across space and modality in working memory
Source: Mem Cognit. 2025 Oct 17;54(4):1183–99. doi: 10.3758/s13421-025-01804-y (PMC13253784; doi:10.3758/s13421-025-01804-y)
Supplement: Supplementary file 1 — Supplementary file1 (DOCX 1289 kb) [file 13421_2025_1804_MOESM1_ESM.docx]

Supplementary materials

Since we examined the effect of prioritization on different binding types and assigned different values to the serial positions, we additionally conducted a three-way ANOVA (a 2 [priority condition] × 2 [binding type] × 4 [serial position] ANOVA) to explore the interaction between these three factors within the same analysis.

Experiment 1

The 2 (priority condition) × 2 (binding type) × 4 (serial position) repeated measures ANOVA indicated no main effect of prioritization (Priority-SP1 M = 0.56, SE = 0.01; No-priority M = 0.55, SE = 0.02; (F(1,34) = 0.80, p = .378, $\eta_{p}^{2}$= 0.02; BF_10_ = 0.18). A main effect of serial position emerged (Greenhouse-Geisser corrected F(1.97, 67.04) = 36.32, p < .001, $\eta_{p}^{2}$ = 0.52; BF_10_ > 10,000). Pairwise comparisons (corrected using Bonferroni – Holm) revealed significant differences between SP1 (M = 0.47, SE = 0.03) and SP3 (M = 0.61, SE = 0.02; p < .001; BF_10_ > 1000), SP1 and SP4 (M = 0.72, SE = 0.03; p < .001; BF_10_ > 10,000), SP2 (M = 0.41, SE = 0.02) and SP3 (p < .001; BF_10_ > 10,000), SP2 and SP4 (p < .001; BF_10_ > 10,000), SP3 and SP4 (p = .002; BF_10_ > 10,000). A main effect of binding type emerged, with higher accuracy in the unitized (M = 0.58, SE = 0.02) than spatially separated (M = 0.52, SE = 0.01), (F(1,34) = 14.86, p <.001, $\eta_{p}^{2}$= 0.30; BF_10_ = 13.72).

There was a significant interaction between SP and prioritization (Greenhouse-Geisser corrected F(2.55, 86.69) = 4.93, p = .005, $\eta_{p}^{2}$ = 0.13; BF_10_ = 10.18) and between SP and binding type (Greenhouse-Geisser corrected F(2.85, 96.95 = 4.03, p = .011, $\eta_{p}^{2}$ = 0.11; BF_10_ = 2.96). There was no significant interaction between binding type and prioritization (F(1, 34) < 0.01, p = .981, $\eta_{p}^{2}$ < 0.01; BF_10_= 0.18); or for the three-way interaction between SP, binding type and prioritization (F(3,102) = 0.82, p = .487, $\eta_{p}^{2}$ = 0.02, BF_10_ = 0.12).

Experiment 2

The 2 (priority condition) × 2 (binding type) × 4 (serial position) repeated measures ANOVA indicated no main effect of prioritization (Priority-SP1 M = 0.57, SE = 0.02; No-priority M = 0.55, SE = 0.02; (F(1,34) = 0.59, p = .450, $\eta_{p}^{2}$ = 0.02; BF_10_ = 0.18) or binding type (F(1,34) = 0.52, p = .474, $\eta_{p}^{2}$ = 0.02; BF_10_ = 0.19). A main effect of serial position emerged (Greenhouse-Geisser corrected F(2.13, 72.47) = 41.29, p < .001, $\eta_{p}^{2}$ = 0.55; BF_10_ > 10,000). Pairwise comparisons (corrected using Bonferroni – Holm) revealed significant differences between SP1 (M = 0.50, SE = 0.02) and SP4 (M = 0.76, SE = 0.02; p < .001; BF_10_ > 10,000), SP2 (M = 0.45, SE = 0.02) and SP3 (M = 0.53, SE = 0.02; p = .030; BF_10_ = 81.02), SP2 and SP4 (p < .001; BF_10_ > 10,000), SP3 and SP4 (p < .001; BF_10_ > 10,000).

There was a significant interaction between SP and prioritization (Greenhouse-Geisser corrected F(2.38, 80.86) = 16.52, p < .001, $\eta_{p}^{2}$ = 0.33; BF_10_ > 10,000) and between SP and binding type (Greenhouse-Geisser corrected F(2.38, 80.81 = 3.38, p = .031, $\eta_{p}^{2}$ = 0.09; BF_10_= 0.90). There was no significant interaction between binding type and prioritization (F(1, 34) = 0.01, p = .919, $\eta_{p}^{2}$ < 0.01; BF_10_= 0.16); or for the three-way interaction between SP, binding type and prioritization (F(3,102) = 0.86, p = .464, $\eta_{p}^{2}$ = 0.03, BF_10_ = 0.16).

Excluded experiment

One experiment was removed from the main study due to a methodological timing issue, where the auditory and visual stimuli in the cross-modal condition were somewhat asynchronous. This made interpretation of the outcomes unclear, and thus the experiment did not clearly contribute positively to the work. However, we still found it useful to include it as supplementary material.

Method

Participants

Thirty participants (aged 18-22 years; M = 19.23; SD = 1.04; 25 females and 5 males) took part in this experiment in exchange for course credit. They were all native English speakers, and none reported a history of neurological disorders. The participants had normal or corrected-to-normal vision and no color blindness. Informed consent was acquired in accordance with the guidelines set by the University of Leeds's Psychology Ethics Committee (Ethics reference number: PSYC-608).

Design and Procedure

The method was similar with Experiment 2, with the same material set, design, and trial procedure. The only exception was that stimulus exposure duration was 1000ms for the visual item. However, as auditory stimuli were naturally voiced at between 450-600ms in duration, participants were therefore exposed to visual stimuli for 1000ms, while they were exposed to auditory stimuli for 450-600ms. This created an asynchrony between visual and auditory stimuli, and an imbalance in exposure time between the unitized and cross-modal conditions. Thus, this experiment was excluded from the main body of the paper.

As with Experiment 2, a 2 x 2 repeated measures design was implemented in each experiment, with two types of binding type condition (unitized and cross-modal binding) and two types of prioritization condition (priority-SP1 and no-priority).

Results

Effect at SP1 (Targeted SP)

Figure 7 shows mean performance at serial position 1 in the binding and priority conditions, along with the mean difference in performance between priority-SP1 and no-priority. A 2 (Priority: Priority SP1 vs no-priority) x 2 (Binding type: unitised vs cross-modal) repeated-measures ANOVA revealed a marginally null main effect of prioritization (F(1,29) = 4.02, p = .054,  $\eta_{p}^{2}$ = 0.12; BF_10_ = 1.46) with weak Bayesian support, and no effect of binding type (F(1,29) = 0.03, p = 0.870, $\eta_{p}^{2}$ < .001; BF_10_ = 0.25). There was also no interaction between prioritization and binding type (F(1,29) = 2.32, p = .138, $\eta_{p}^{2}$ = 0.07; BF_10_= 0.71), indicating that there was no improvement in prioritized item in priority condition compared to no priority condition and there was no performance difference between binding conditions.


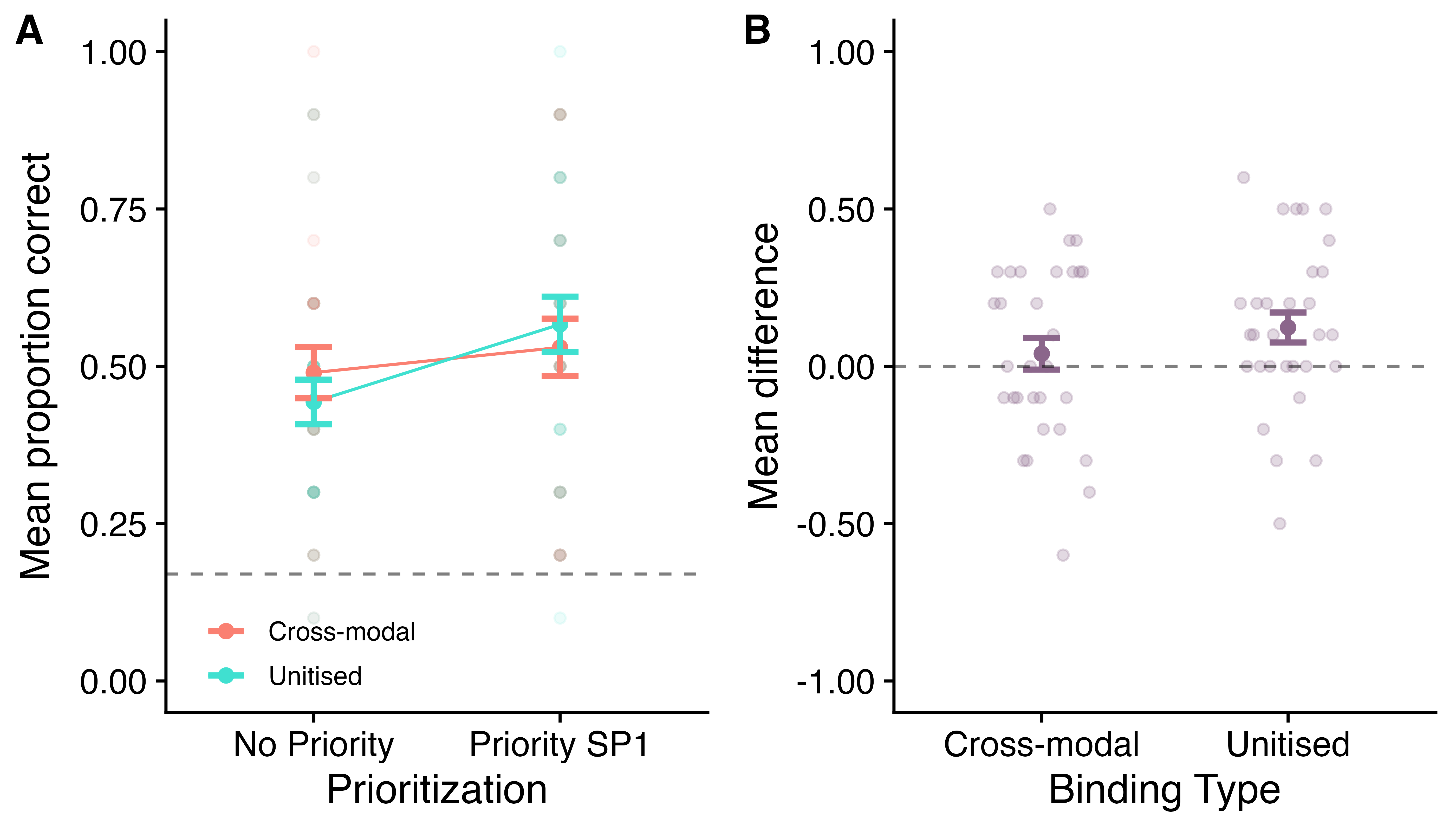


Figure 7. A. Mean performance at serial position 1 (the targeted SP) for the priority and binding conditions. B. Mean difference between priority-SP1 and no-priority conditions at serial position 1 for each binding type. Values above 0 indicate higher performance in the priority-SP1 condition. Error bars show SE and light dots show individual participants.

Effects on less valuable SPs (2-4)

Figure 8 shows mean performance averaged across low value serial positions (2-4) in the binding and priority conditions, along with the mean difference in performance between priority-SP1 and no-priority. A 2 (Priority: Priority SP1 vs no-priority) x 2 (Binding type: unitised vs separated) repeated-measures ANOVA revealed no main effect of prioritization(F(1,29) = 2.57, p = .119, $\eta_{p}^{2}$ = .08; BF_10_ = 0.78) or binding (F(1,29) = 0.03, p = .860, $\eta_{p}^{2}$ = .001; BF_10_= 0.24). There was also no significant interaction between prioritization and binding type (F(1,29) = 0.07, p = .798, $\eta_{p}^{2}$ = .002; BF_10_ = .27).


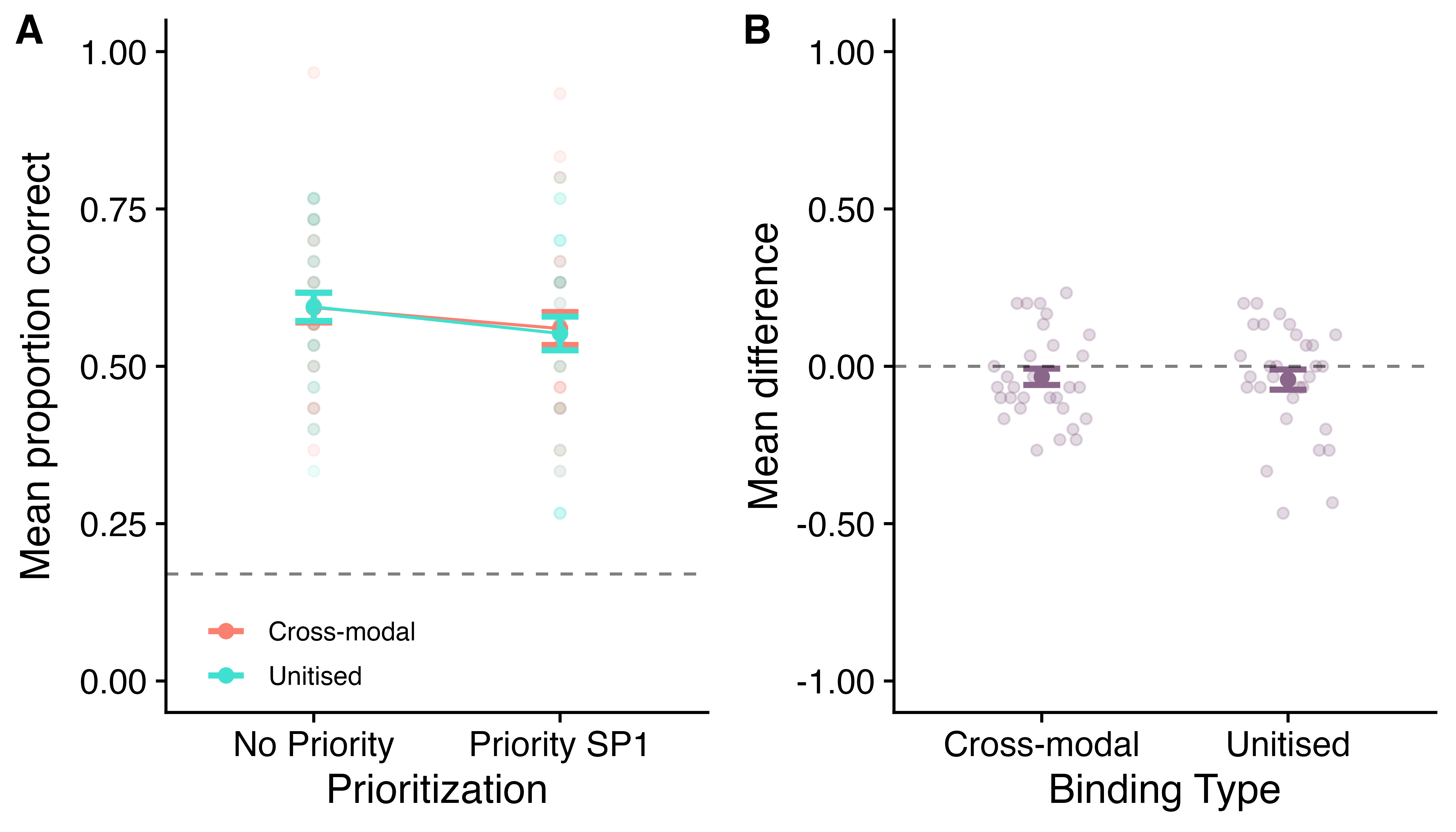


Figure 8. A. Mean performance averaged across low value serial positions (2-4) for the priority and binding conditions. B. Mean difference between priority-SP1 and no-priority conditions for each binding type. Values below 0 indicate lower performance in the priority-SP1 condition. Error bars show SE and light dots show individual participants. Here, “Priority-SP1” refers to the experimental condition in which SP1 was assigned high value, but the data in the panels reflect the low-value positions (SP2–SP4).

Overall effects of priority and binding conditions

Finally, we examined whether overall performance (across all serial positions) varied with binding condition and prioritization. A pair of paired-sample t-tests was conducted, comparing unitized vs. cross-modal binding, and Priority-SP1 vs. No-Priority. As predicted, there was no effect in either t-test, with no significant difference between unitized (M = .56, SE = .02) and cross-modal binding (M = .56, SE = .02) (t(29) = -0.23, p = .817, BF_10_ = 0.20, d = -0.04), or between priority-SP1 (M = .55, SE = .02) and no priority conditions (M = .56, SE = .02) (t(29) = -0.46, p = .651, BF_10_ = 0.21, d = -0.08).
